# Nitric Oxide Enables Germination by a Four-Pronged Attack on ABA-Induced Seed Dormancy

**DOI:** 10.3389/fpls.2018.00296

**Published:** 2018-03-09

**Authors:** Santiago Signorelli, Michael J. Considine

**Affiliations:** ^1^The UWA Institute of Agriculture, The University of Western Australia, Perth, WA, Australia; ^2^The School of Molecular and Chemical Sciences, The University of Western Australia, Perth, WA, Australia; ^3^UWA School of Agriculture and Environment, The University of Western Australia, Perth, WA, Australia; ^4^Departamento de Biología Vegetal, Universidad de la República, Montevideo, Uruguay; ^5^Irrigated Agriculture, Department of Primary Industries and Regional Development, South Perth, WA, Australia; ^6^Centre for Plant Sciences, School of Biology, University of Leeds, Leeds, United Kingdom

**Keywords:** nitric oxide, dormancy, post-translational regulation, plant development, abscisic acid (ABA), phytohormone crosstalk

## Abstract

Nitric oxide (⋅NO) is known to attenuate dormancy and promote germination, a function that seemingly depends on crosstalk with the abscisic acid (ABA) signaling network. In the past 2 years, a number of independent studies have revealed that ⋅NO gates the ABA signaling network at multiple steps, ensuring redundant and effectively irreversible control of germination. Here we summarize the recent studies, and propose a model of the multiple functions of ⋅NO in seed dormancy.

## Introduction

Elemental nitrogen is capable of a range of oxidation states (-3 to +5). The formation of reactive nitrogen species (RNS) is thus a necessary consequence of nitrogen metabolism. These RNS include peroxynitrite (ONOO^-^), and the free radicals ⋅NO and nitrogen dioxide (⋅NO_2_). Due to their high reactivity, RNS can modify the structure and function of proteins through the nitration of tyrosine or nitrosylation of cysteine residues. These functions have been adopted as central modes of post-translational regulation, governing wide developmental, acclimation and stress response processes in plants. For example, root and shoot elongation, pollen and seed development, stomatal closure, and antioxidant defense (Prado et al., 2004; Lombardo et al., 2006).

Among the RNS, ⋅NO is the most well-studied, and several developmental and adaptive functions have been assigned. Distinct roles of ⋅NO in regulating seed dormancy and germination have been described, including the interaction with other plant growth regulators (Beligni and Lamattina, 2000; Batak et al., 2002; Bethke et al., 2006). Nevertheless, the collective influence of ⋅NO is pervasive, demonstrating function in tropic growth responses, root development and branching, nodule formation, cell wall lignification, xylem differentiation, cellulose biosynthesis, stomatal aperture, pollen tube growth, floral transitions, fruit maturation, and leaves senescence (reviewed by Sanz et al., 2014). Moreover, at physiological concentrations, ⋅NO is in the gas phase and able to diffuse across membranes, and may have relatively long half-life. These features make ⋅NO an important local and long-range signaling molecule and gasotransmitter (Lamattina and García Mata, 2016).

## Rns Effect Post-Translational Control of Phytohormone Signaling

Many of the developmental functions of RNS result from the interference with phytohormone signaling pathways, mainly by the *S*-nitrosylation of key intermediate signaling proteins.

For example, the *S*-nitrosylation of phosphotransfer proteins functions in the repression of cytokinin (CK) signaling (Feng et al., 2013). In a similar way, the *S*-nitrosylation of OPEN STOMATA1 (OST1) and SUCROSE NON-FERMENTING1 (SNF1)-RELATED PROTEIN KINASE2.6 (SnRK2.6) negatively regulates ABA signaling in guard cells (Wang et al., 2015a). Other SnRK2 proteins are also susceptible to *S*-nitrosylation, attenuating ABA control of seed germination (Wang et al., 2015b). In addition, ⋅NO was shown to increase the DELLA protein concentration, which negatively regulates gibberellic acid (GA) signal transduction (Lozano-Juste and Leon, 2011; Krasuska et al., 2016). Together, these observations demonstrate that ⋅NO can fine-tune phytohormone signaling at several levels, and is thus an important sensory medium.

## Involvement of Rns in the Aba-Mediated Dormancy Control

**FIGURE 1:**
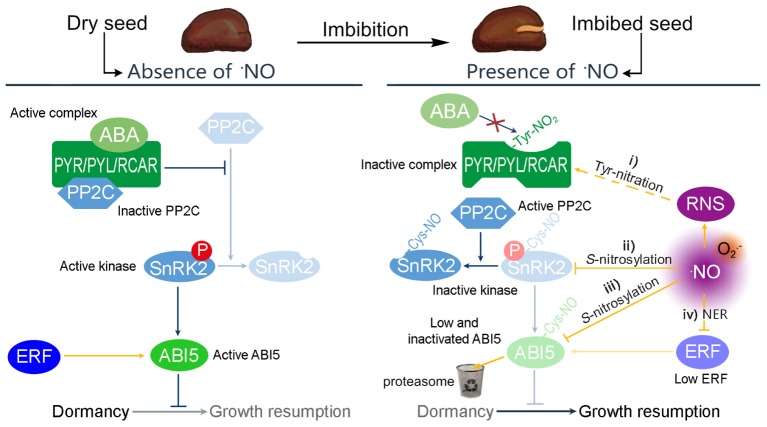
Possible mechanisms by which RNS modulate the ABA regulation of dormancy. In absence of ⋅NO, the transcription factor ABI5 controls the expression of genes relevant to ensure the dormant state. The expression of *ABI5* is induced by the Group VII ETHYLENE RESPONSE FACTORS (ERF) and the activity of ABI5 is promoted by SnRK2 kinases. ABA binds to the PYR/PYL/RCAR receptor to complex PP2C and avoids the inactivation of SnRK2. However, seed imbibition produces an increase of ⋅NO levels, resulting in a potential increase of different RNS. In this situation, the ABA control of dormancy can be attenuated by four different pathways: (i) the PYR/PYL/RCAR complex can be *S*-nitrosylated avoiding the interaction with ABA; (ii) ⋅NO can *S*-nitrosylates SnRK2 kinases inactivating their kinase activity; (iii) ⋅NO can target ABI5 to the proteasome by *S*-nitrosylation affecting the expression of genes under its regulation; and (iv) ⋅NO also targets the Group VII ERF to the proteasome, via the N-end rule pathway of proteolysis.

The ABA network governing seed dormancy is well-described (Graeber et al., 2012). In this network, the binding of ABA to the ABA receptors, PYR/PYL/RCAR, results in the inactivation of type 2C protein phosphatases (PP2C). This inactivation triggers the action of the SnRK2 kinase, which promotes the activity of the basic leucine zipper transcription factor ABSCISIC ACID INSENSITIVE5 (ABI5) (**Figure 1**). In turn, ABI5 exerts considerable transcriptional control over dormancy (Skubacz et al., 2016). ABI5 is thus considered a key repressor of seed germination and post-germination development (Finkelstein and Lynch, 2000; Lopez-Molina et al., 2001).

Crosstalk between ⋅NO and ABA has been demonstrated by pharmacological and genetic approaches, for example the enhanced dormancy potential and ABA hypersensitivity of ⋅NO-deficient seeds of arabidopsis (*Arabidopsis thaliana*; Lozano-Juste and Leon, 2010), which was later explained by the hyperaccumulation of ABI5 (Albertos et al., 2015). However, on closer inspection it is clear that RNS can interfere with ABA signaling by four independent pathways (**Figure 1**).

Firstly, RNS can inactivate the PYR/PYL/RCAR receptor by tyrosine-nitration (Castillo et al., 2015), enabling the activity of PP2C, which inactivates SnRK2. Thus, the influence of ABI5 is attenuated (**Figure 1**, i). Secondly, different SnRK2 proteins (SnRK2.6, SnRK2.2, and SnRK2.3) were shown to be inactivated by *S*-nitrosylation (by ⋅NO), affecting ABA signaling not only in stomatal closure but also seed germination (Wang et al., 2015b). As mentioned above, in a nitrosative condition, most of the available SnRK2 would be dephosphorylated (inactive). It seems clear, however, that any remaining phosphorylated SnRK2 can be inactivated directly by ⋅NO, which nitrosylates a cysteine residue near the kinase catalytic site, blocking the kinase activity (**Figure 1**, ii; Wang et al., 2015a). Thirdly, ⋅NO assists the degradation of ABI5 by the *S*-nitrosylation at cysteine-153, targeting it to the proteasome by enhancing its interaction with CULLIN4-based and KEEP ON GOING E3 Ligases (**Figure 1**, iii; Albertos et al., 2015). Accordingly, the levels of ⋅NO and the amount of *S*-nitrosylated proteins increase in barley seed embryos during the first hours post-imbibition (Ma et al., 2016). Finally, ⋅NO promotes the degradation of the Group VII ETHYLENE RESPONSE FACTORS (ERF, **Figure 1**, iv) via the N-end rule pathway of proteolysis. These ERFs are positive regulators of the transcription of *ABI5*, and hence their degradation limits further synthesis (Gibbs et al., 2014).

## Perspectives

• RNS can modulate a single signaling pathway at multiple levels. Here we have described the fine-tuning of ABA signaling by four independent mechanisms, all of which apparently negatively regulate the canonical ABA pathway. RNS crosstalks with other phytohormone pathways have been demonstrated. Due to the pervasive influence of RNS activities on enzyme functions, now we expect further detail to emerge on the redundancies of RNS signaling, positive, negative and conflicting influences.
• Although the crosstalk between RNS and ABA is well-developed, questions still remain. For example, whether the nitration of the PYR/PYL/RCAR complex does occur *in vivo*. The influence of ⋅NO is particularly dependent on spatial, temporal and concentration conditions.
• ⋅NO acts as a gasotransmitter affecting diverse biological processes. In plants, there are many pathways of ⋅NO synthesis. However, no ⋅NO synthase has been identified in plants yet. The potential finding of a plant ⋅NO synthase would be key to manage the ⋅NO homeostasis and thus the processes under its regulation.
• From a management point of view, and in particular for seed producers, it would be interesting to develop procedures to manage endogenous ⋅NO levels. This would lead to the possibility of producing seeds with prolonged or reduced dormancy, as desired.
• With the arising of genome editing techniques, it would be possible to replace susceptible residues to nitration and nitrosylation by amino acids with similar physicochemical characteristics in order to reduce the susceptibility of the enzymes to RNS but keeping their functionality.

## Concluding Remarks

Nitric oxide participates in the regulation of the dormancy release by (i) the tyrosine nitration of ABA receptors, (ii) *S*-nitrosylation of SnRK2s, (iii) *S*-nitrosylation of ABI5, and (iv) the degradation of ERF. This evidence supports an inverse molecular link between ⋅NO and ABA hormone signaling in which ⋅NO acts upstream and downstream.

## Author Contributions

MC and SS jointly conceived and wrote the manuscript.

## Conflict of Interest Statement

The authors declare that the research was conducted in the absence of any commercial or financial relationships that could be construed as a potential conflict of interest.
