# Corrigendum: Nitric Oxide Enables Germination by a Four-Pronged Attack on ABA-Induced Seed Dormancy

**DOI:** 10.3389/fpls.2018.00654

**Published:** 2018-05-17

**Authors:** Santiago Signorelli, Michael J. Considine

**Affiliations:** ^1^The UWA Institute of Agriculture, The University of Western Australia, Perth, WA, Australia; ^2^The School of Molecular and Chemical Sciences, The University of Western Australia, Perth, WA, Australia; ^3^UWA School of Agriculture and Environment, The University of Western Australia, Perth, WA, Australia; ^4^Departamento de Biología Vegetal, Universidad de la República, Montevideo, Uruguay; ^5^Irrigated Agriculture, Department of Primary Industries and Regional Development, South Perth, WA, Australia; ^6^Centre for Plant Sciences, School of Biology, University of Leeds, Leeds, United Kingdom

**Keywords:** nitric oxide, dormancy, post-translational regulation, plant development, abscisic acid (ABA), phytohormone crosstalk

There was an unnecessary arrow in Figure 1 as published. The correct version of Figure 1 appears below. The author's apologies for the mistake, which may have led to misinterpretation. This error and correction does not affect the interpretation or intent of the figure with respect to the role of nitric oxide-dependent regulation of seed dormancy and germination.

**Figure 1:**
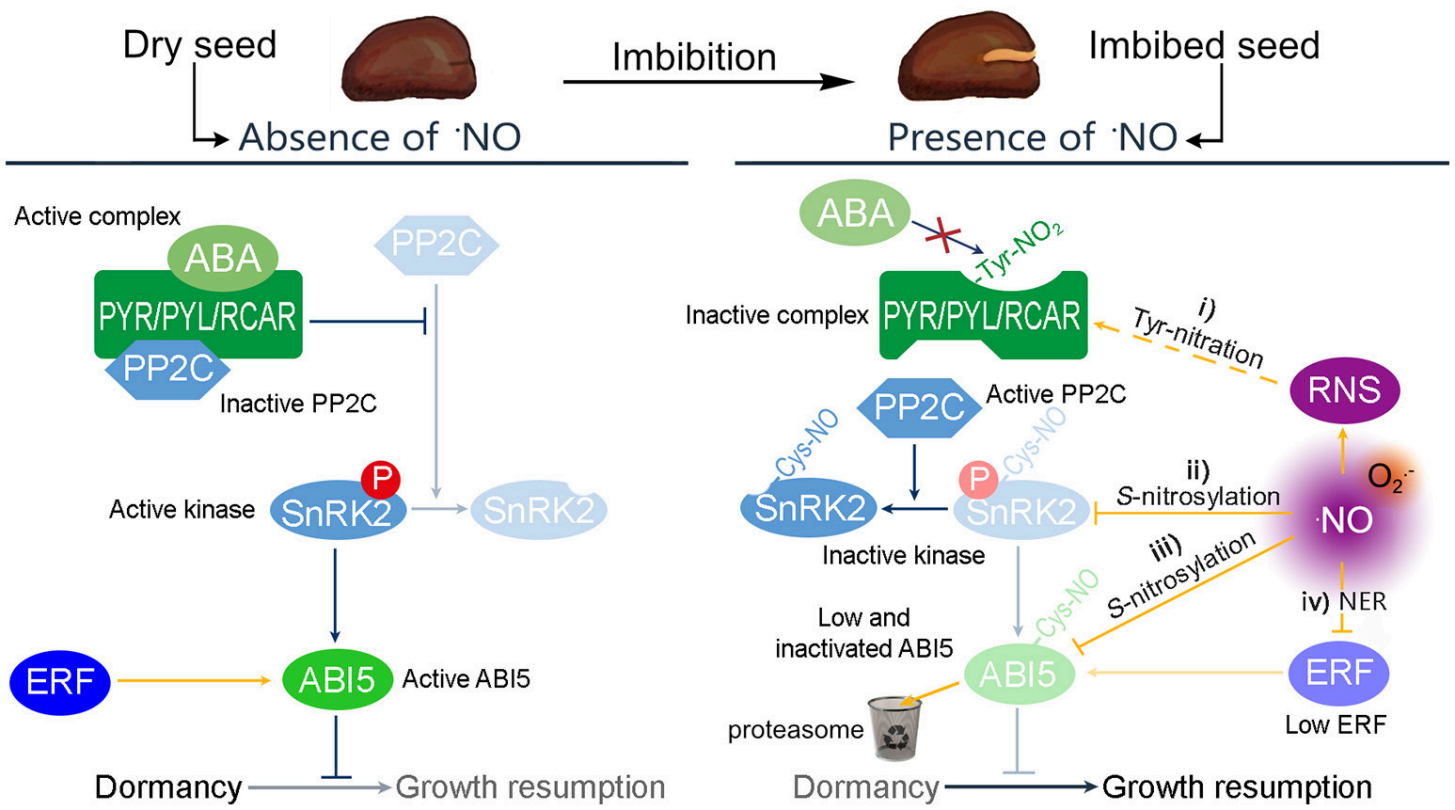
Possible mechanisms by which RNS modulate the ABA regulation of dormancy. In absence of ·NO, the transcription factor ABI5 controls the expression of genes relevant to ensure the dormant state. The expression of *ABI5* is induced by the Group VII ETHYLENE RESPONSE FACTORS (ERF) and the activity of ABI5 is promoted by SnRK2 kinases. ABA binds to the PYR/PYL/RCAR receptor to complex PP2C and avoid the inactivation of SnRK2. However, seed imbibition produces an increase of ·NO levels, resulting in a potential increase of different RNS. In this situation, the ABA control of dormancy can be attenuated by four different pathways: (i) the PYR/PYL/RCAR complex can be *S*-nitrosylated avoiding the interaction with ABA; (ii) ·NO can *S*-nitrosylate SnRK2 kinases inactivating their kinase activity; (iii) ·NO can target ABI5 to the proteasome by *S*-nitrosylation affecting the expression of genes under its regulation; and (iv) ·NO also targets the Group VII ERF to the proteasome, via the N-end rule pathway of proteolysis.

The original article has been updated.

## Conflict of interest statement

The authors declare that the research was conducted in the absence of any commercial or financial relationships that could be construed as a potential conflict of interest.

